# Carotid Intima-Media Thickness and Cardiovascular Risk in Adolescents with Overweight or Obesity: Are There Gender-Related Differences?

**DOI:** 10.31083/j.rcm2510352

**Published:** 2024-09-29

**Authors:** Christian Saleh

**Affiliations:** ^1^Basel, Switzerland

**Keywords:** ultrasound, carotid artery, surrogate marker, carotid intima-media thickness

Dear Sir,

Di Bonito *et al*. [1] published in the recent issue of Reviews in Cardiovascular Medicine a study titled, “Sex-Related Differences in Cardiovascular Risk in Adolescents with Overweight or Obesity”. The authors wrote, “Pediatric obesity is closely associated with cardiometabolic comorbidities, but the role of sex in this relationship is less investigated” [1]. The study aimed to analyze the gender-related differences on cardiometabolic risk factors and preclinical signs in overweight/obese (OW/OB) adolescents [1]. The cross-sectional study included 988 subjects with OW/OB, ranging in age between 10–18 years old [1]. As surrogate marker for pre-clinical atherosclerosis the authors used the carotid intima-media thickness (CIMT) [1]. For 107 subjects (59 males and 48 females) sonographic data were available [1]. The authors measured the CIMT at the near and far walls of the right and left common carotid arteries (CCA) in proximity of the bifurcation [1, 2]. The mean value of these 4 maximum thicknesses was reported as carotid IMT [1, 2]. The authors concluded, that “this cross-sectional study conducted in adolescents with OW/OB confirmed our hypothesis that sex-related differences in cardiovascular risk might be present in adolescents with OW/OB” [1]. Some comments are needed to evaluate the results of this study in a more balanced way. Recommendations as to CIMT measurement protocols are variable [3, 4]. Di Bonito *et al*. [1] missed discussing the different recommended measurement protocols [3, 4] and within this context the limitations in regards to CIMT as surrogate marker. There are two main recommendations measuring the CIMT [3, 4]: one is to perform a single-site measure at the CCA at the far wall, for higher spatial resolution [4], while the second recommendation is to perform multiple-site carotid artery (CA) measurements, e.g. including far/near walls of different CA sections (CCA, bifurcation and internal CA) [3]. This latter procedure is argued to capture more accurately the asymmetric presentation of atherosclerosis [3]. A single-site measurement may coincide with a normal segment, but still within an atherosclerotic vessel. Moreover, Di Bonito *et al*. [1] did not address another critical procedural aspect in CIMT measurement, namely whether measurement was synchronized with the cardiac cycle; recommended is to perform CIMT measurement at the end-diastolic phase [4, 5]. Carotid intima-media thickness (CIMT) varies, due to vessel diameter changes, during the cardiac cycle. Differences in CIMT between systole and diastole (on average 0.041 mm) were reported [5]. Authors, who do not synchronize with the cardiac cycle, overlook the fact that the categorization of patients in normal and abnormal CIMT groups is based on solely sub-millimetric differences. Minimal inaccuracies make here therefore the difference. These inaccuracies, in CIMT estimation may be even greater in the Di Bonito *et al*. [1] study due to their manual CIMT measurements [2], ultimately sufficient to translate into the significant sub-millimetric differences between the two groups. Given these critical methodological issues and considering that the reported CIMT values for both genders are within the normal range and devoid of any diagnostic as prognostic value (male 0.52 mm ± 0.06, female 0.49 mm ± 0.07, *p* value = 0.011), the conclusions, within the context of the CIMT data of this study [1], should be analyzed with caution.

## Availability of Data and Materials

Not applicable.
